# Comparative Planning of Flattening-Filter-Free and Flat Beam IMRT for Hypopharynx Cancer as a Function of Beam and Segment Number

**DOI:** 10.1371/journal.pone.0094371

**Published:** 2014-04-10

**Authors:** Yvonne Dzierma, Frank G. Nuesken, Jochen Fleckenstein, Patrick Melchior, Norbert P. Licht, Christian Rübe

**Affiliations:** Department of Radiation Oncology, Saarland University Medical Center, Homburg/Saar, Germany; University of California Davis, United States of America

## Abstract

Although highly conformal dose distributions can be achieved by IMRT planning, this often requires a large number of segments or beams, resulting in increased treatment times. While flattening-filter-free beams offer a higher dose rate, even more segments may be required to create homogeneous target coverage. Therefore, it is worthwhile to systematically investigate the dependence of plan quality on gantry angles and number of segments for flat vs. FFF beams in IMRT planning. For the practical example of hypopharynx cancer, we present a planning study of flat vs. FFF beams using three different configurations of gantry angles and different segment numbers. The two beams are very similar in physical properties, and are hence well-suited for comparative planning. Starting with a set of plans of equal quality for flat and FFF beams, we assess how far the number of segments can be reduced before the plan quality is markedly compromised, and compare monitor units and treatment times for the resulting plans. As long as a sufficiently large number of segments is permitted, all planning scenarios give good results, independently of gantry angles and flat or FFF beams. For smaller numbers of segments, plan quality decreases both for flat and FFF energies; this effect is stronger for fewer gantry angles and for FFF beams. For low segment numbers, FFF plans are generally worse than the corresponding flat beam plans, but they are less sensitive to a decrease in segment number if many gantry angles are used (18 beams); in this case the quality of flat and FFF plans remains comparable even for few segments.

## Introduction

Since the advent of modern treatment planning techniques such as intensity modulated radiotherapy (IMRT), highly conformal radiotherapy treatment can be achieved with simultaneous good coverage of the planning target volume (PTV) and adequate sparing of organs at risk (OAR). This often comes at the cost of an increase in treatment time. Even disregarding the impact on the clinical schedule, treatment times longer than a few minutes are uncomfortable for the patients and carry an increased risk of intra-fraction motion [1], which may compromise plan quality especially when narrow PTV and OAR margins are used as in modern image-guided radiotherapy (IGRT).

A reduction of treatment time can be achieved in three ways. First, using fewer beams or segments in an IMRT plan implies the risk of losing plan conformality. Second, high-end modern treatment techniques such as volume modulated arc therapy (VMAT) and RapidArc offer high-quality plans as good as IMRT (or better if IMRT is restricted to a low number of segments or beams), with generally much faster treatment times ([2]; see [3] for a review). Even so, locations such as head-and-neck tumours generally require more than one gantry rotation or hybrid fields [4]–[5], which again increases treatment time; furthermore, these advanced treatment techniques are not commonly available. Third, flattening-filter-free (FFF) beams can be applied in both IMRT and VMAT treatment planning, with the practical advantage of much higher dose rates as compared with normal flat beams [6]. A draw-back to be considered here is that the conical beam profile of FFF beams is generally found to require more segments and/or more monitor units to achieve the same standards of PTV homogeneity than for flat beams (e.g., [7]–[8]); this again lengthens treatment time somewhat, particularly for large PTVs. The greatest reduction in treatment time may arise from a combination of arc treatment with FFF beams; however, here we will consider FFF beams in IMRT treatment planning because of its rapidly spreading availability.

The aim of this work is to systematically investigate the dependence of plan quality on gantry angles and number of segments for flat vs. FFF beams in IMRT planning for the practical example of hypopharynx cancer. We start with an IMRT solution using 70 segments distributed between 7 and 18 gantry angles, for which we determine inversion objectives and constraints that create near-identical plans for the flat and FFF beams of the Siemens Artiste (Siemens Healthcare, München, Germany). The two beam lines – flat 6 MV (6X) and FFF 7 MV (7XU) – are very similar in physical properties, such as mean energy, depth-dose-curve and surface dose [9], and are hence well-suited for comparative planning. Starting from these plans, we assess how far the number of segments can be reduced before the plan quality is markedly compromised, and compare monitor units and treatment times for the resulting plans. The questions of relevance are:

1. Which composition of gantry angles is best?
2. Does the 7XU energy offer comparable plan quality to the 6× beam, and how does this depend on gantry angles?
3. How do the answers to these two questions respond to a reduction in segment number?

This study is organized as follows: We start with a standard IMRT plan with 7 gantry angles, which used to be a standard approach at our institution at the beginning of this study. Normally, less than 70 segments would have been used to reduce treatment times; here, they are chosen so as to inflict no restriction on the plan quality owing to an insufficient number of segments – this way, an inversion not restricted by segment number should be achieved. After finding a set of inversion parameters that produces equally good plans with 6× and 7XU beams for this plan template, these inversion objectives and constraints (i.e. dose and DVH objectives/constraints used in the optimization) are used throughout this study. In a second step, plans are calculated for both energies, each for 7, 11 or 18 beams, and reducing the segments stepwise from 70 to 25. We then investigate comparatively how the choice of gantry angles, segments and energy (flat vs. FFF) influences the plan quality.

## Patients and Methods

### 1. Patient collective

#### Ethics statement

Eight patients (five male, three female; 44–77 years of age, mean age 55 yr) with head and neck cancer, in whom adjuvant radiation or simultaneous radiochemotherapy was indicated, were examined. The patients, treated between October 2010 and September 2012, were selected from a previous planning study in our department examining the impact of an individually fabricated oral distance applicator for external beam irradiation on dose reduction in oral in head and neck cancer (located in the oral cavity, oro-/hypopharynx or larynx (Fleckenstein et al., in prep.)). This previous study was approved by the local ethics committee (Aerztekammer des Saarlandes) and all patients gave written informed consent for subsequent scientific studies. For the present study, the anonymized data from the previous study were used, with no further interaction with the patients.

Regardless of the real location of the patients' tumours contours were established for hypothetical uniform tumour sites (floor of mouth, oropharynx and hypopharynx).

**Figure 1:**
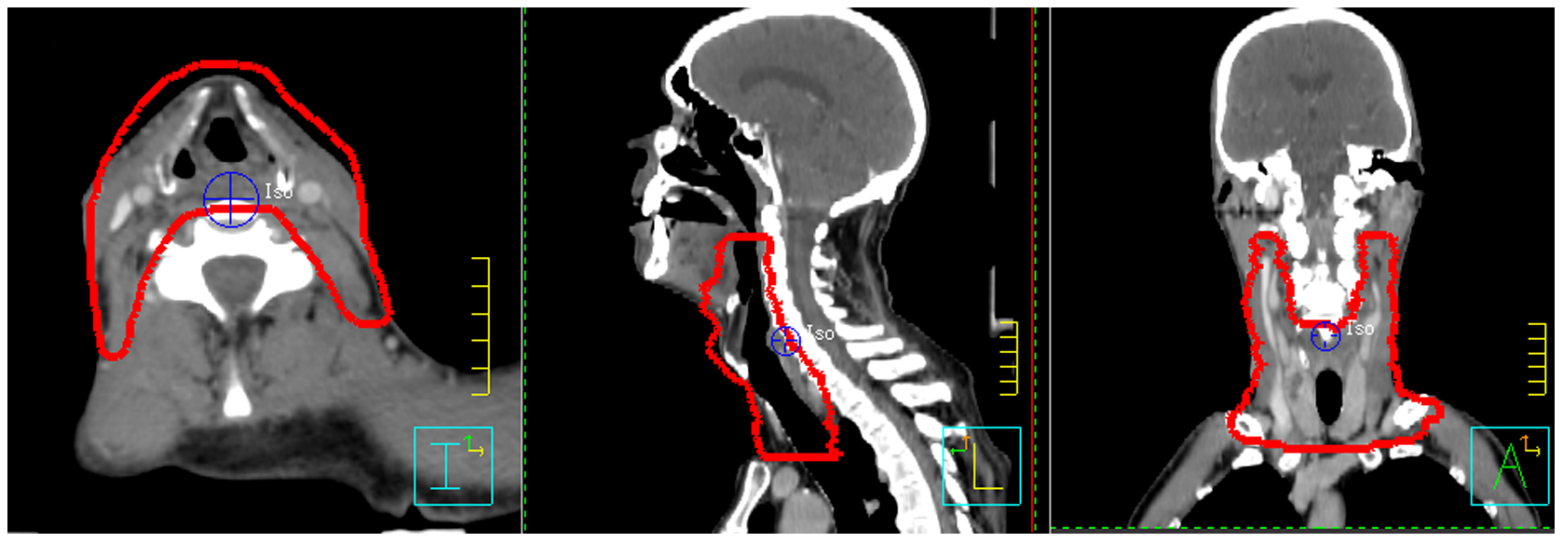
Example PTV with isocenter, transverse (left), sagittal (center) and coronal views (right).

For the present study all plans are based on the PTV of the hypopharynx cancer site including bilateral cervical and supraclavicular lymph nodes (level II–V, see Fig. 1), while boost-contours were disregarded. A reference dose of 50 Gy was prescribed to the isocenter, administered in 2 Gy fractions.

### 2. Treatment planning

Treatment planning was performed with the Philips Pinnacle^3^ treatment planning system V9.2 and 9.4 based on CT data from Philips Brilliance CT BigBore (Philips Healthcare, Amsterdam, the Netherlands). IMRT inversion was done using direct machine parameter optimisation (DMPO). The final dose distribution was calculated with a collapsed cone algorithm on a dose grid of 0.4 cm resolution.

Three different gantry configurations were used: a “simple“ IMRT plan with 7 beams, an improved IMRT plan with 11 beams, and a multiple-beam plan intended to simulate rotational treatments (18 beams) – see Table 1 for a summary of plan characteristics.

**Table 1:** Plan characteristics and IMRT inversion parameters for three different beam arrangements.

	7 beams	11 beams	18 beams
**Gantry angles (collimator angle)**	0° (90°), 50° (0°), 80° (0°), 150° (90°), 210° (90°), 280° (0°), 310° (0°)	0° (90°), 30° (0°), 65° (0°), 100° (0°), 135° (90°), 170° (90°), 190° (90°), 225° (90°), 260° (0°), 295° (0°), 330° (0°)	0° (0°), 20° (0°), 40° (0°) 60° (0°), 80° (0°), 100° (0°), 120° (0°), 140° (0°), 160° (0°), 180° (0°), 200° (0°), 220° (0°), 240° (0°), 260° (0°), 280° (0°), 300° (0°), 320° (0°), 340° (0°)
**Max. # segments**	70	70	70
**Min. segment**	Area: 7 cm^2^, MU: 5		
**Min. lamella**	Pairs: 2, distance: 1.5 cm		
**Isocentre**	Automatically placed inside PTV, then manually shifted to front edge of spine		

The first part of this study aimed at finding a set of inversion objectives/constraints which can be used equally well for both 6× and 7XU plans, so as to provide a straightforward “recipe“ to be used in planning. This seemed mandatory for the standardization and comparibility of plans for both beam lines. This approach was facilitated since the Pinnacle^3^ TPS uses a gradient-based inversion method, so that two sets of similar constraints will reproducibly lead to very similar plans. For a subset of 3 patients, different sets of inversion objectives and constraints were tested for a plan configuration with 7 beams and 70 segments, the standard head-and-neck IMRT setting at our institution at the time the study started. We started with the optimized ‘in-house’ objectives and constraints used for 6× beams. When good 6× plans were created, the same template was optimized for the 7XU energy. We modified our choice of inversion objectives/constraints for the first three patients iteratively by trial-and-error until good plans were achieved for both 6× and 7XU for these three patients; these objectives/constraints were then maintained for all eight patients and all beam and segment scenarios for the rest of the study.

**Figure 2:**
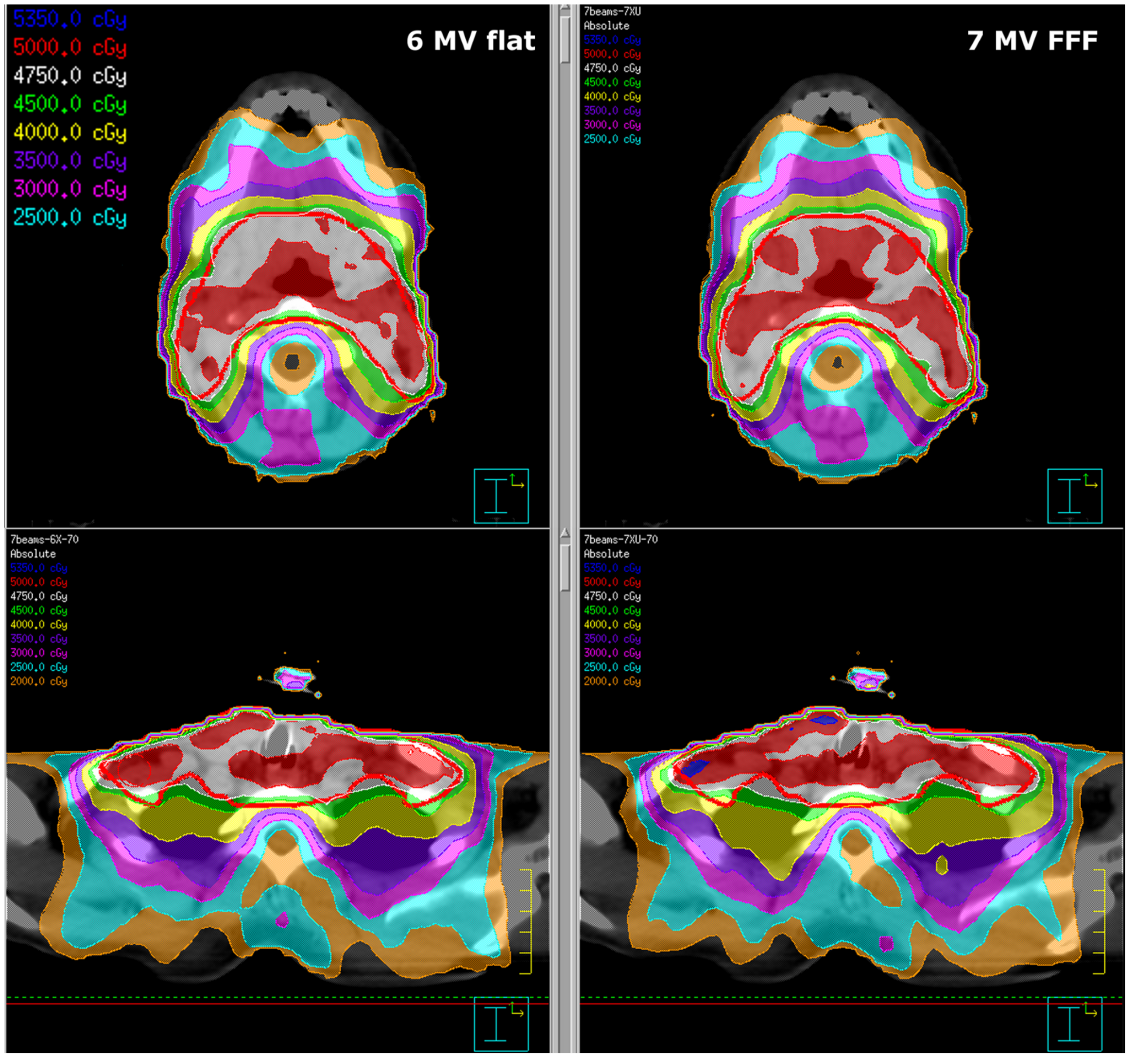
Example head & neck plan using 7 beams, 6 MV (left) vs. FFF 7 MV (right).

**Figure 3:**
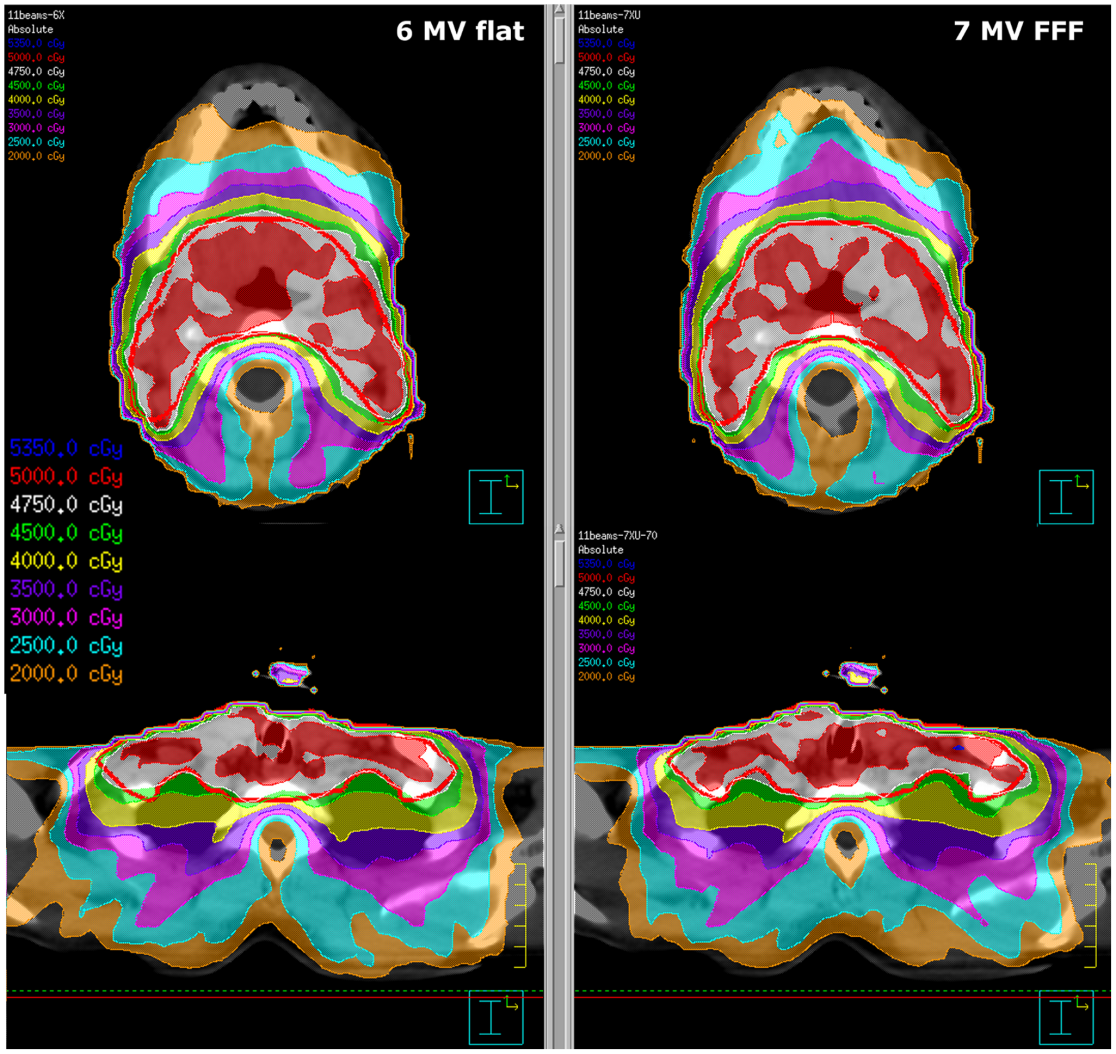
Example head & neck plan using 11 beams, 6 MV (left) vs. FFF 7 MV (right), same patient as in Figure 2.

Comparing a number of different choices of inversion objectives/constraints, it was found that most objectives routinely used at our institution for 6× plans could be applied for the 7XU beam. The main difference between the resulting plans was a reduced PTV homogeneity for the FFF plans, which required the inclusion of an additional constraint forcing 5% PTV homogeneity. This constraint hardly influenced the 6× plans, which generally complied with it even when it was not explicitly stated (in fact it is included in the PTV maximum, minimum and uniform dose objectives). Since our aim was to find a set of objectives/constraints which could be applied equally well to the 6× and 7XU beams, the homogeneity constraint was included. The final choice of objectives/constraints which yielded plans of sufficient quality is given in Table 2; example dose distributions are shown in Figures 2–3. Different choices of objectives/constraints may certainly be used to create good quality plans; we here present one example which we use at our institution to reliably yield adequate plans for most patients.

**Table 2:** Final optimized inversion constraints.

Structure	Objective	Weight
PTV*	Uniform dose 50 Gy (100%)	20
	Max dose 50.42 Gy	20
	Min dose 48.75 Gy (95%)	20
	Homogeneity 5%	constraint
	Min DVH 95% 47.5 Gy	constraint
	Max DVH 5% 50.25 Gy	constraint
PTV-Ring (+ 3 mm to +7 mm)	Max dose 45 Gy (90%)	20
External without PTV (+10 mm)	Max dose 40 Gy (80%)	20
	Max dose 25 Gy (50%)	1
Spinal cord	Max dose 33.33 Gy	20
	Max dose 25 Gy	1
Parotis (if not inside PTV)	Max DVH 30% 14.17 Gy	20

*exluding parotis where only edge of parotis reaches into PTV.

**Figure 4:**
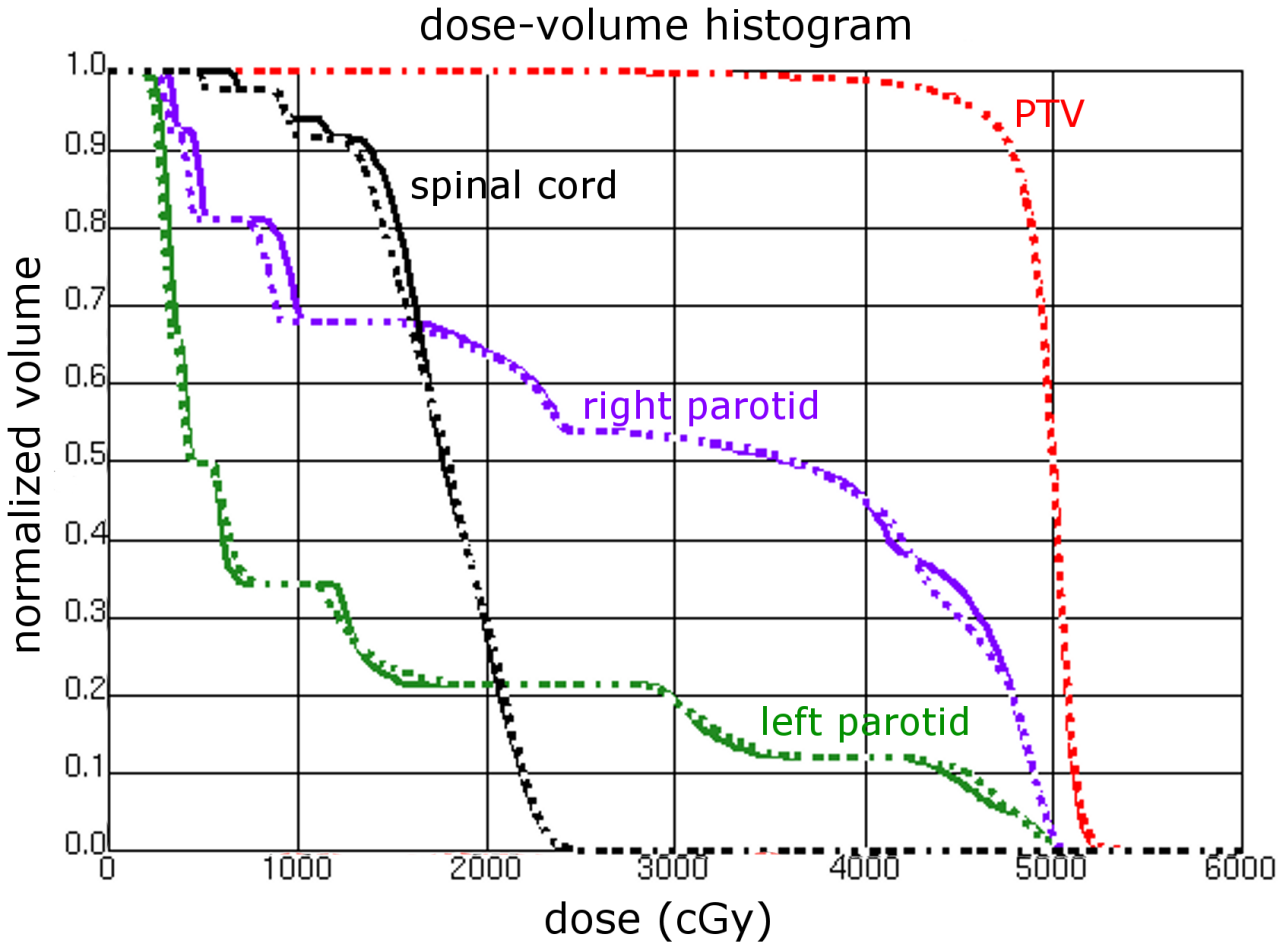
Dose-volume histogram of plans shown in Figure 3. Solid line: 6 MV, dashed line: FFF 7 MV. The right parotid fell inside the PTV, so it received considerable doses as compared with the left parotid, which was spared as much as possible.

Using this choice of constraints, for each patient three different gantry and collimator configurations were used (for both energies, respectively): the “simple“ IMRT plan with 7 beams, an improved IMRT plan with 11 beams, and the 18 beam plan intended to simulate rotational treatments. All plans were revised by an experienced radiation oncologist and were considered clinically acceptable. A representative DVH of a starting plan with 11 beams is shown in Figure 4.

Given the “starting“ plans judged of sufficient quality, the number of segments allowed in the optimization was reduced from 70 to 25 in a number of steps (50, 40, 35, 30, 25) for each of the plan varieties. This resulted in 36 different plan scenarios, counting all beam arrangements, energies, and segment numbers.

### 3. Plan evaluation

The following measures of plan quality were considered [10]–[12]: Paddick's conformity index
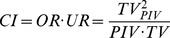



as the product of the overdose ratio OR and underdose ratio UR, where
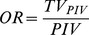



relates the volume of the PTV included in the prescription isodose (TV_PIV_) to the total prescription isodose volume PIV  =  V(95%) and 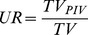



relates the target volume inside the prescribed isodose to the total PTV volume (TV).

PTV homogeneity is measured by the homogeneity index



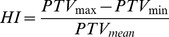
.

The dose fall-off is given by the gradient index



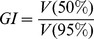
.

Together with the quality indices, maximum dose to the spinal cord, mean parotid dose and maximum dose in the PTV are considered. Taken together, these values should give a good grasp both on PTV coverage and dose outside the PTV. The evaluation of plan quality based on the DVH, in particular dose to organs at risk, is based on the QUANTEC recommendations [13]–[15].

### 4. Statistical analysis

When individual plan scenarios were compared (e.g., 7 beams, 70 segments, 6× vs. 7XU), a normal distribution was assumed and T-test for paired data was used; when pooled plans were compared (e.g., all plans using 6× vs. all plans with 7XU), a normal distribution could not be presumed, and Wilcoxon's signed-rank test of paired data was used. A 5% level of significance was applied. The overall comparison across all plans was performed by the Friedmann test and one-way ANOVA, which was performed in case of equal variances, a prerequisite which was checked using the Brown-Forsythe test.

## Results

### 1. 7 beams IMRT plans using flat and FFF beams with 70 segments

When comparing the two beam energies for the initial planning with 7 beams and 70 segments, the visual inspection of the dose distribution shows that both sets of plans are clinically acceptable for all patients, with very similar quality (for an example, see Fig. 2). For both beam modalities, there is no statistically significant difference in all the quality measures considered (CI, HI, GI, PTV max, parotid mean, spinal cord max). Both beam energies provide good quality plans with the same choice of inversion parameters, which are henceforth retained for the rest of the study.

### 2. Influence of gantry angles and segment number reduction on plan quality for flat and FFF beams

#### a) Plans with 70 segments

**Figure 5:**
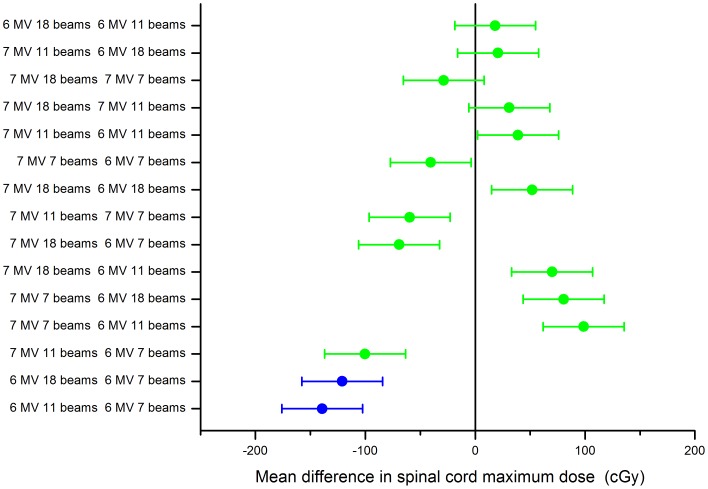
Result of Holm-Bonferroni-test for spinal cord maximum dose of plan scenarios with 70 segments. Plotted are the mean differences; significant deviations from zero are blue, non-significant values are green.

In a first step, plans with 7, 11 and 18 gantry angles were compared with each other for 6× and 7XU, again starting with 70 segments. All six scenarios were compared by ANOVA (having checked by the Brown-Forsythe test that the prerequisite of equal variances is met). No significant differences are found with respect to CI, GI, HI, mean parotid dose D_mean_(parotid) and PTV maximum dose D_PTV_(max). Only the maximum dose to the spinal cord D_max_(spinal cord) is improved in the 6×, 11 beam and 18 beam plans relative to the 7 beam plan, but all other differences are not significant (Figure 5). In particular, no significant difference is observed in the quality indices of 6× vs. 7XU plans of equal beam arrangement. It hence appears that all plan scenarios (both energies and all three gantry scenarios) offer good results, as long as the segment number is sufficiently high.

From visual analysis, the 11 beam arrangement offers slightly improved plan quality over the 7 beam scenario in most cases, both from the point of view of PTV coverage and sparing of organs at risk and tissue outside the PTV (e.g., Figs. 2–3). Moving to 18 beams, the quality is sometimes improved (in particular for 6×), sometimes reduced (mostly for 7XU) relative to 11 beams, but usually better than for the 7 beam arrangement. Even in cases where the 18 beam plans are better than the 11 beam plans, the improvement is – at best – of marginal clinical relevance. In a clinical setting, the shorter treatment time for the 11 beam plans would always have resulted in a decision to treat with these plans. Depending on the patient, either the 6× or 7XU plans (11 beams) are preferred – in all cases, the differences are minor.

#### b) Reduction of segment number

**Figure 6:**
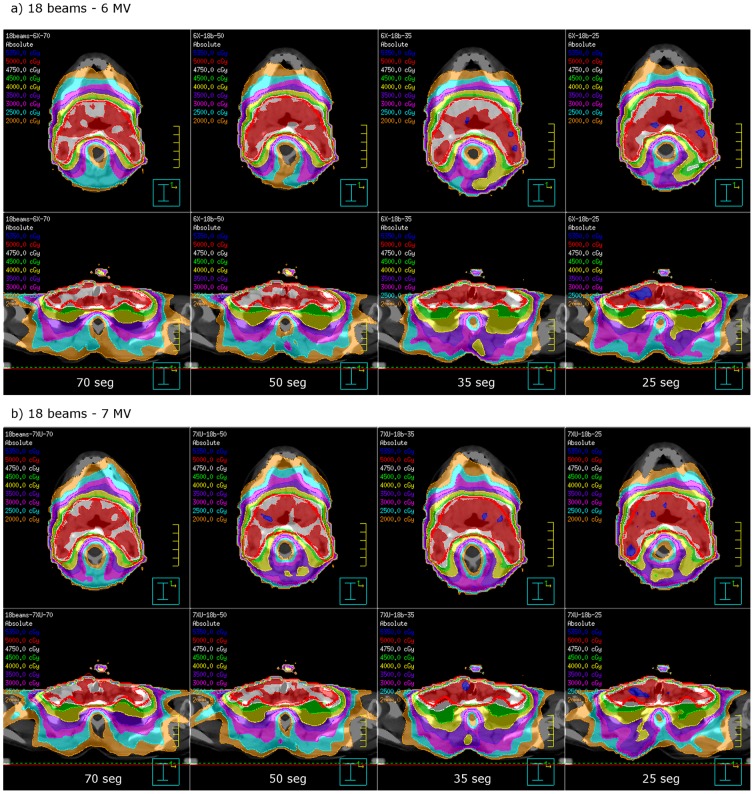
Example head & neack plan using 18 beams, for decreasing segment number (70 to 25 segments), 6 MV (a) vs. FFF 7 MV (b), same patient as in Figure 2.

By visual comparison the plan quality decreases with a lower segment number (see Fig. 6 for an example). Evidently, the 70 segment plans are superior. In many cases, the difference from the 50 segment plan is only minor, in some cases a somewhat worse dose distribution results from the 50 segment plans – however, all 50 segment plans are still very good. 40 segments plans are always clearly worse than the 70 segment plans. In a number of patients, these plans would still be acceptable, although generally the maximum is higher, PTV coverage worse, and dose outside the PTV higher (e.g., larger V(80%), sometimes reaching behind the spinal cord). These effects become more evident for smaller segment numbers. Here, variations between patients increase – some patient plans are still acceptable down to 25 segments, while others are already unacceptable starting at 35 (or even 40) segments, depending both on the patient, energy, and choice of gantry angles.

**Figure 7:**
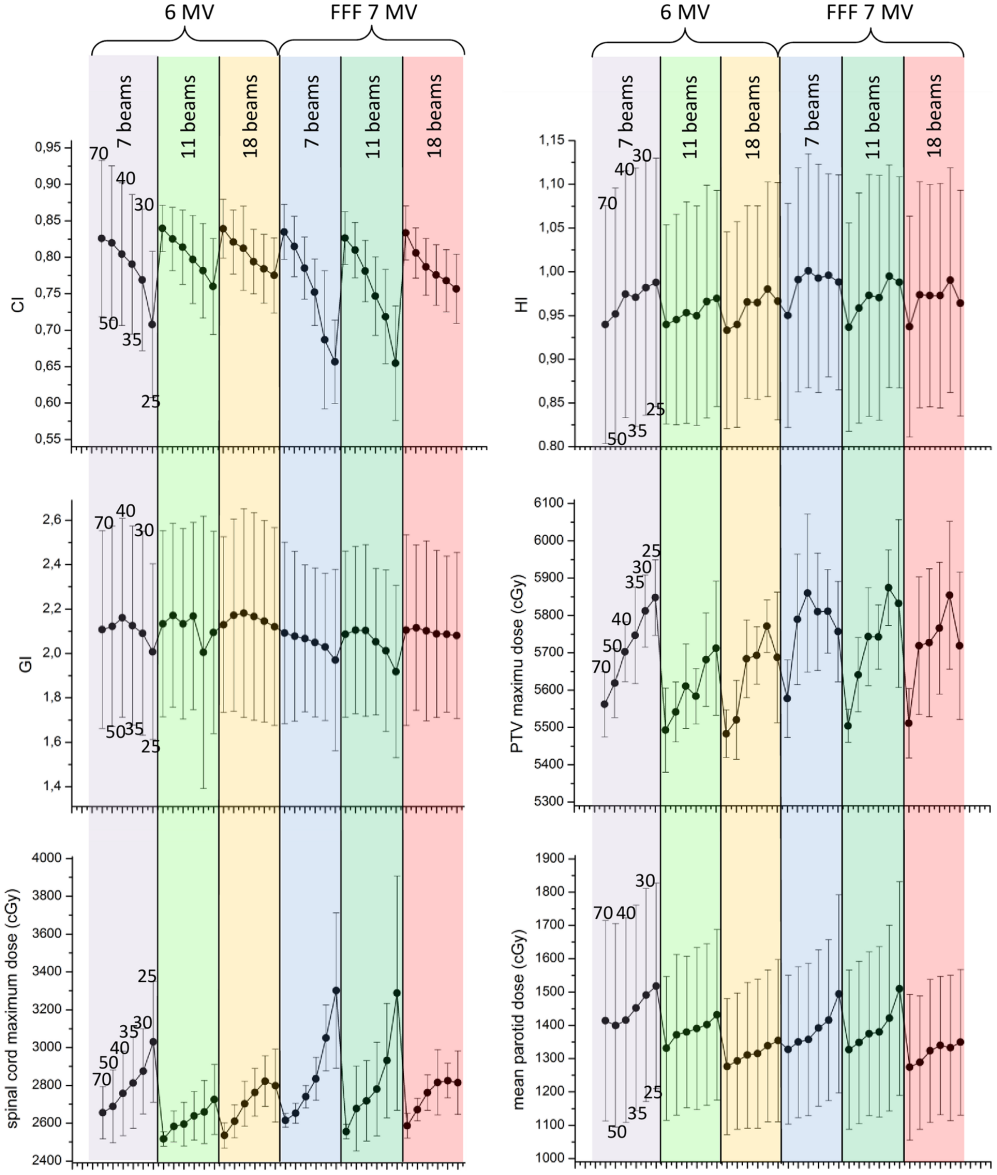
Quality measures and doses to organs at risk for all plan scenarios, plotted with standard deviations. CI: conformity index, HI: homogeneity index, GI: gradient index. Within each plan scenario, segment number decreases from left to right (shown for 6 MV, 7 beams).

To investigate these effects systematically and quantitatively, the quality measures are considered. The Brown-Forsythe test finds no significant differences in the variances for all measures of quality except D_max_(spinal cord), so one-way ANOVA can be performed. In homogeneity and gradient index, the plan versions do not show significant differences, although the gradient index appears to decrease slightly with a lower segment number. The conformity index systematically decreases with lower segment number for all scenarios, and significant differences are found between plan versions, which are analyzed in more detail in the following. The change in quality measures with segment number for the different plans is displayed in Figure 7.

To compare plans between scenarios, we applied the Tukey test to rank the plans according to CI and find plans that can be grouped together (Table 3). This ranking and grouping can only give a first tentative estimate of plan quality, but we use it as a first step to call the three tiers of plans “good quality”, “medium quality” and “poor quality”, for comparison with the other measures of quality. In fact, this grouping confirms the visual impression that all 70 segment plans and most 50 segment plans, together with few 40 segment scenarios, give good results, whereas plans with 25 to 35 segments generally perform poorly. A similar grouping could have been obtained by simple “cut off” values on CI. Our grouping corresponds to values of around 0.81 and 0.78; a simpler choice might be 0.8 and 0.75, which would place all plans with 50 and 70 segments, together with the three 40 segment 6× plans in the “good” group and the six worst CI plans in the “poor” group.

**Table 3:** Plan ranking and grouping according to CI by Tukey test.

6 MV 11 beams 70 seg	0.8395 ± 0.0318	good
6 MV 18 beams 70 seg	0.8391 ± 0.0405	good
7 MV 7 beams 70 seg	0.8348 ± 0.0379	good
7 MV 18 beams 70 seg	0.8335 ± 0.0370	good
7 MV 11 beams 70 seg	0.8263 ± 0.0363	good
6 MV 7 beams 70 seg	0.8259 ± 0.1073	good
6 MV 11 beams 50 seg	0.8250 ± 0.0435	good
6 MV 18 beams 50 seg	0.8209 ± 0.0439	good
6 MV 7 beams 50 seg	0.8196 ± 0.1059	good
7 MV 7 beams 50 seg	0.8148 ± 0.0415	good
6 MV 11 beams 40 seg	0.8138 ± 0.0509	good
6 MV 18 beams 40 seg	0.8123 ± 0.0578	good
7 MV 11 beams 50 seg	0.8098 ± 0.0380	good
7 MV 18 beams 50 seg	0.8059 ± 0.0344	medium
6 MV 7 beams 40 seg	0.8043 ± 0.0979	medium
6 MV 11 beams 35 seg	0.7969 ± 0.0605	medium
6 MV 18 beams 35 seg	0.7939 ± 0.0442	medium
6 MV 7 beams 35 seg	0.7904 ± 0.0958	medium
7 MV 18 beams 40 seg	0.7868 ± 0.0390	medium
7 MV 7 beams 40 seg	0.7851 ± 0.0426	medium
6 MV 18 beams 30 seg	0.7840 ± 0.0473	medium
6 MV 11 beams 30 seg	0.7820 ± 0.0648	medium
7 MV 11 beams 40 seg	0.7811 ± 0.0421	medium
7 MV 18 beams 35 seg	0.7758 ± 0.0414	poor
6 MV 18 beams 25 seg	0.7751 ± 0.0516	poor
6 MV 7 beams 30 seg	0.6890 ± 0.0972	poor
7 MV 18 beams 30 seg	0.7679 ± 0.0476	poor
6 MV 11 beams 25 seg	0.7600 ± 0.0658	poor
7 MV 18 beams 25 seg	0.7566 ± 0.0473	poor
7 MV 7 beams 35 seg	0.7520 ± 0.0455	poor
7 MV 11 beams 35 seg	0.7466 ± 0.0537	poor
7 MV 11 beams 30 seg	0.7185 ± 0.0646	poor
6 MV 7 beams 25 seg	0.7076 ± 0.1003	poor
7 MV 7 beams 30 seg	0.6868 ± 0.0947	poor
7 MV 7 beams 25 seg	0.6568 ± 0.0572	poor
7 MV 11 beams 25 seg	0.6548 ± 0.0787	poor

The following plans are grouped together by the Tukey test:

from 6 MV 11 beams 70 seg to 7 MV 11 beams 35 seg,

from 6 MV 18 beams 70 seg to 7 MV 11 beams 30 seg,

from 7 MV 11 beams 70 seg to 6 MV 7 beams 25 seg,

from 7 MV 18 beams 50 seg to 7 MV 7 beams 30 seg,

from 7 MV 18 beams 35 seg to 7 MV 7 beams 25 seg,

from 6 MV 18 beams 25 seg to 7 MV 11 beams 25 seg,

from 7 MV 11 beams 30 seg to 7 MV 11 beams 25 seg.

**Figure 8:**
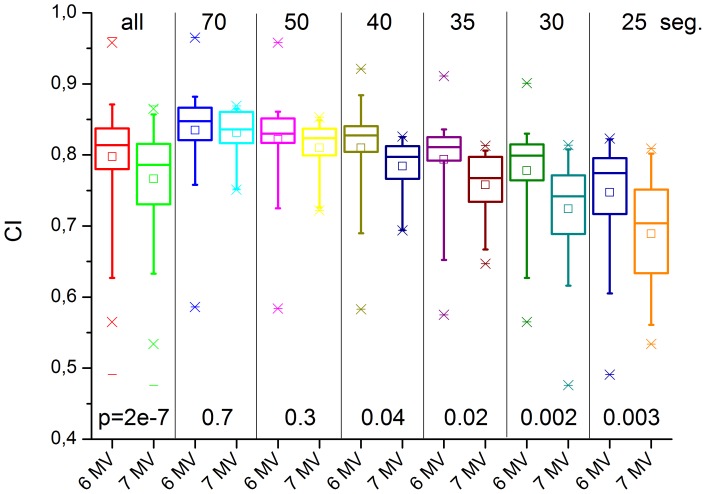
Box-diagram of CI from pairwise test of 6 MV vs. FFF 7 MV, taking all scenarios (7, 11, or 18 beams) together, for all plans (all) or a given number of segments (given in top line).

No matter how the groups are defined, the relative amount of 7XU plans appears to increase in the lower-quality groups – a hypothesis we test by pooling all plans with 6× and 7XU, respectively: each 6× plan, across all patients, beam arrangements and segment numbers, is compared with the corresponding 7XU plan using Wilcoxon's signed-rank test of paired data. The result is highly significant for worse overall performance of the 7XU plans (p  =  2e-12). This means that over all planning scenarios, the 7XU results appear significantly worse as compared with 6×, although we have seen that this is not the case for 70 segment plans. We check where the quality difference between 6× and 7XU plans becomes significant by performing paired T-tests for the combined scenarios (7, 11, 18 beams) segment-wise (Fig. 8). We find no significant difference between the 6× and 7XU plans for 70 and 50 segments, but significant differences for fewer segments. This is physically understandable, since the FFF beam may need more segments to achieve uniform PTV coverage – the plan quality is hence impaired if only few segments (40 or less) are permitted.

This phenomenon is also visually apparent in Fig. 7, where the decrease in CI with lower segment number is evidently more pronounced for 7XU plans than for the 6× plans. This is why the 7XU plans achieve comparable quality for high segment numbers, but fall short at lower segment numbers. An exception appears to be the 18 beam scenario, which is considerably better than 11 or 7 beams in the case of 7XU, and does not show such a strong decline in quality with lower segment numbers. From the point of view of CI values, best plans for low segment numbers are achieved with 6×, 11 or 18 beams, or 7XU, 18 beams. A similar behavior is found for the maximum dose to the spinal cord and the mean parotid dose. This is somewhat surprising, since we expected the 18 beam plans to cope worse with few segments, given the fact that the individual field modulation is reduced when a small number of segments is distributed on many beams. For the 18 beam plans at 25 segments, only 7 beams can be intensity modulated at all, the majority having just a single segment. Still, the dose distribution (both conformity index and dose to organs at risk) is improved in these plans.

**Figure 9:**
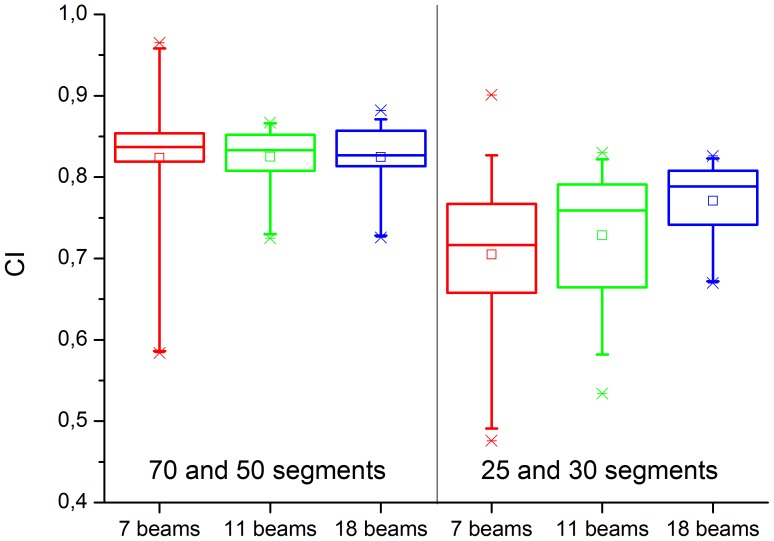
Testing the three gantry scenarios (7, 11, or 18 beams) against each other for both energies combined, for high segment numbers (left) vs. low segment numbers (right). No significant difference is found for the three scenarios with 70 and 50 segments. For 25 and 30 segments, no significant difference is seen between plans with 7 and 11 beams. The 18 beam plan is significantly higher in CI than both the 7 and 11 beam plans (p  =  1.38 e-6 and p  = 0.0106, respectively).

**Figure 10:**
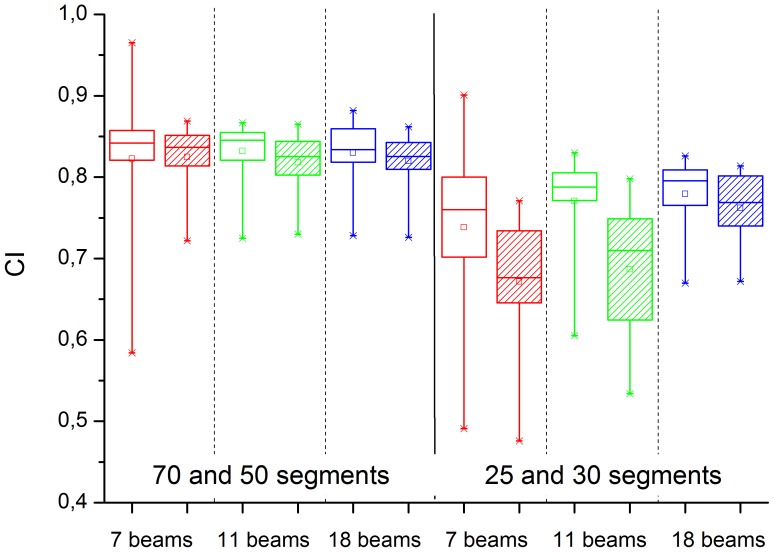
Testing the three gantry scenarios (7, 11, or 18 beams) against each other for flat 6 MV (empty boxes) and FFF 7 MV (filled boxes), separately, for high segment numbers (left) vs. low segment numbers (right).

This visual result is again checked statistically by paired testing of the 7, 11 and 18 beam plans for small segment numbers. In a first step, we take together the 6× and 7XU plans. For 70 and 50 segments, no significant difference is found between the gantry angle scenarios; for 30 and 25 segments, the 18 beam plans are significantly better than the 7 and 11 beam plans (Fig. 9). To separate the effects of energy and gantry angles, we test 6× and 7XU separately (Fig. 10). For 30 and 25 segments, the 6× plans with 11 and 18 beams are both significantly better in CI than the 7 beam plans (p  =  0.037 and p  =  0.019), but do not differ from each other. For 7XU, the 7 and 11 beam plans are not significantly different from each other, but both inferior as compared with the 18 beam plan (p  =  6.1 e-6 and p  =  0.017). The 18 beam plans cope best with the reduced number of segments, remaining of comparable quality to the 6× plans even when the 7 and 11 beam plans have become considerably worse.

### 3. Summary of results – Plan quality

For a large number of segments (70 or 50, in our case), different gantry angle arrangements can be used to create good quality plans, with no notable difference in quality between 6× or 7XU energies. However, if the number of segments is reduced, this has more pronounced effects on plans with few gantry directions and with FFF energies. For 6× plans, both 11 and 18 beam plans cope relatively well with reduced number of segments (still with worse plan quality than for larger segment numbers, but better than the 7 beam scenario); at 7XU, only the 18 beam arrangement is relatively stable under a reduction of segment number, remaining of comparable quality to the analogous 6× plans. For 7XU plans with 7 or 11 gantry angles, no less than 40 segments should be used to retain decent quality, comparable to 6× plans.

### 4. Amount of time needed to irradiate the plans

To a first approximation, the irradiation time can be estimated from the number of segments, gantry angles, and monitor units (MU) for a given dose rates, if mean times for MLC movements between segments and gantry movement between angles are assumed. Empirically, we have found that calculating with an average segment time 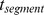 of 7 seconds per segment and a mean gantry time 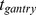 of 13 seconds, the predicted irradiation times agree well with the real irradiation time (deviations usually range below one minute), and rely on this approximation in the evaluation of treatment times. MLC movements are generally faster than gantry rotations, so there is no additional segment time for reaching the first segment of each beam. The formula for the irradiation time hence becomes
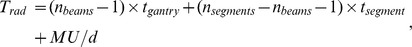
where 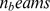 is the number of gantry angles, 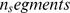 is the number of segments, and *d* is the dose rate. At the Artiste linac, the maximum available dose rate for 6× is 300 MU/min, for 7XU it is 2000 MU/min.

**Figure 11:**
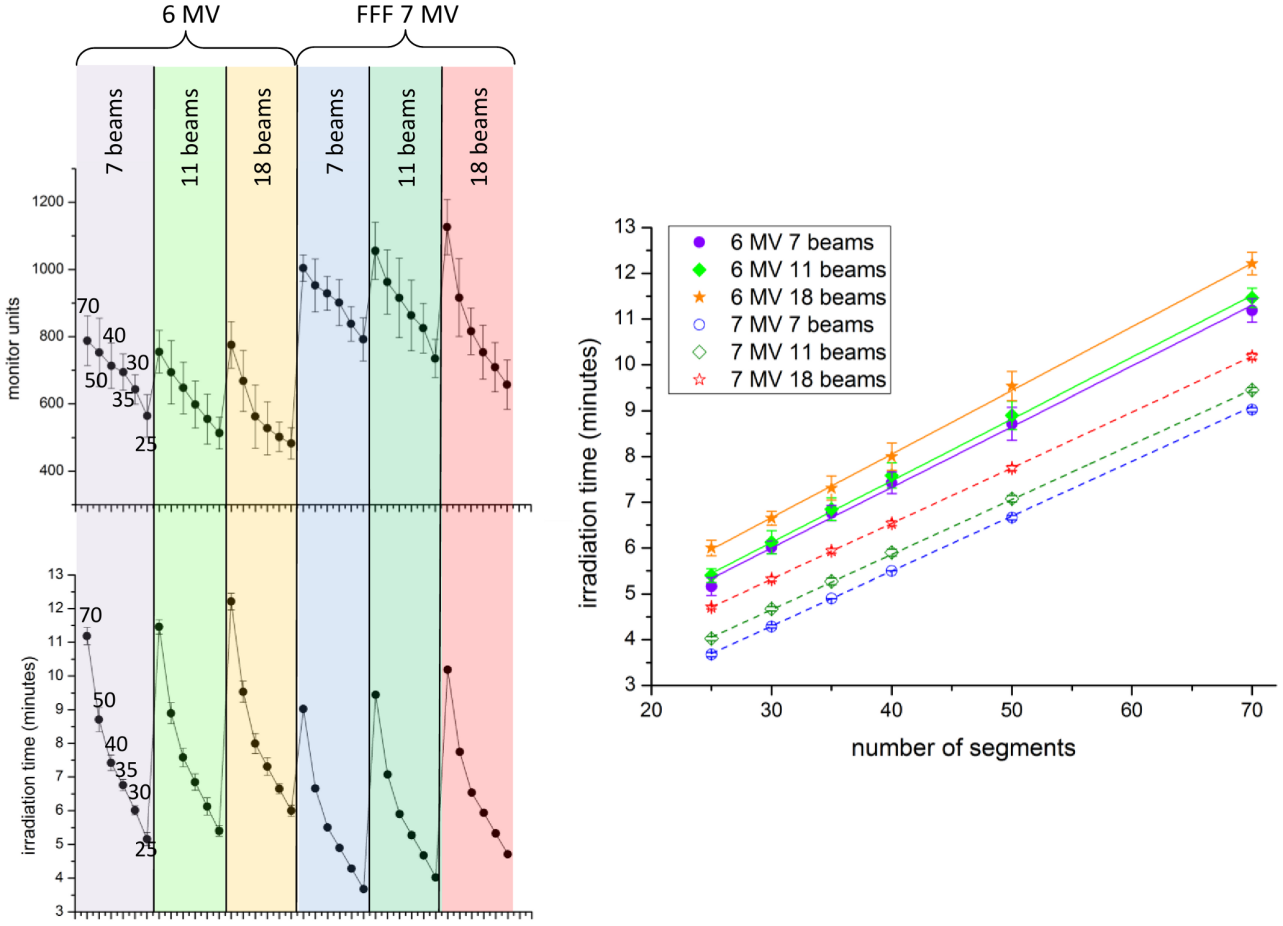
Left: Monitor units and predicted treatment times for different plan scenarios, with standard deviations. Right: linear fit of calculated treatment time as a function of segment number of each scenario.

The number of monitor units needed for the plans is not restricted a priori in the planning. Logically, we find lower MU for fewer segment plans, and more MU for the FFF beam plans, which is concordant with previous studies. Depending on the patient and on the number of segments, 6× plans use between 900 and 450 MU (Figure 11), 7XU plans have 1200 to 550 MU. This increase in MU is compensated by the considerably higher dose rate, so that 7XU plans generally require shorter treatment times (3.5–10 min) as compared with the corresponding 6× plans (5–12 min depending on segment number). In general, fewer gantry angles lead to shorter treatment times for the same number of segments, which is due to the fact that the MLC movement is faster than gantry rotations.

## Discussion

### 1. Planning scenarios

We have presented a sensitivity study of flat and FFF IMRT plans for 7, 11, and 18 beams with respect to a reduction in segment number from 70 to 25. It is well known that plan quality generally decreases with segment number; however, the influence of flat or FFF beams and of beam number has not hitherto been investigated. Including a scenario with 18 beams may appear somewhat unrealistic, since IMRT plans with more than 11 or 13 beams are rarely created. We chose this scenario for two reasons: firstly, to expand the sensitivity analysis to a large number of beams. There is sometimes a tendency in the clinical practice to increase the number of beams while reducing the segment number, choosing, e.g., 13 beams with 33 segments. This study aims to assess how far this idea can be carried, and how FFF beam energies react to this. Secondly, 18 beams are a first step moving towards arc treatment, which was not included in this work. Comparison of the IMRT plans with VMAT would be interesting, but cannot be achieved for the Artiste linac, which is incapable of VMAT. Comparison with a different linac would be biased both by different beam energies and by a different MLC. On the other hand, the closely similar dosimetric characteristics of the flat 6 MV and FFF 7 MV energies are ideal for a planning study, even without VMAT techniques. We therefore included 18 beams as a “tentative arc”, where the plan quality is not limited by a small amount of gantry angles, but benefits from relatively uniform irradiation from beams spaced 20° apart.

It has been proposed that finer grid spacing should be used to reduce discretization errors [16]. For the present context, the great number of plans per patient and large PTV size make a finer spacing hard to handle; besides, many clinics rely on a 4 mm grid in routine patient treatment. Knowing about the limitations, we therefore opted for a 4 mm grid and checked the results for 6 plans per patient to estimate the differences when using a finer spacing of 2.5 mm. Indeed some differences can be discerned in the dose distribution, but they are small compared with the differences between individual plan scenarios. Quality measures are changed less than half the width of inter-patient variation, and do not change the results of the plan comparison.

### 2. Extension of target volume

The present study focusses on hypopharynx cancer, which is an extreme case considering PTV extension in the superior-inferior direction (of the order of 15 cm). This scenario maximizes the influence of the beam flatness. At 10 cm distance from the central axis, the dose of the FFF 7 MV beam has fallen off to around 50% of the maximum, so it is no wonder that the FFF 7 MV plans require more segments and monitor units to achieve good dose coverage of the PTV far from the isocenter. This means the FFF 7 MV plans with few segments will be at a disadvantage as compared with 6 MV plans; at the same time, this effect will become less relevant for smaller target volumes. The conclusions drawn here for the example of hypopharynx cancer will therefore not be valid for small target volumes where flatness effects are reduced: in particular, for the very small field size used in stereotactic treatment, FFF beams can often be applied with hardly any difference from flat beams (compare, e.g., [17]). For large field sizes, however, we expect the results presented in this paper to be representative.

### 3. Treatment time

While short irradiation times (5 minutes or less) can be achieved by using 7XU plans with 25–35 segments and 7 or 11 beams, it must be remembered that these plans are inferior to those with more segments or more beams. The higher number of segments needed by the 7XU plans to achieve plans with adequate quality is compensated by the higher dose rate. For example, the 7XU, 18 beams, 50 segment plan is of comparable quality to the 6X, 7 beam, 40 segment plan, and takes about the same time to treat. Still, most good-quality 7XU plans with, e.g., 50 segments can be irradiated within 6.6 to 7.8 minutes, which is still slightly faster than most 6× plans with 35–40 segments (6.8–8.0 minutes) or more.

### 4. Choice of pre-set inversion objectives

This planning study relies on the application of a pre-defined set of objectives and constraints for the optimization of a number of planning scenarios, with the purpose to ensure comparability between all plans. In the clinical setting, the plans might be further individualized for each patient. However, our aim was not to explore the limits of individual planning capability, but to compare plan quality for different beam arrangements, energies, and segment numbers – this comparison would have been biased if the objectives had been varied and each plan optimized *ad libitum*. In this case, the results would depend on the amount of time and effort the planning physicist or dosimetrist takes for each scenario, which is what we explicitly wanted to avoid.

### 5. Implications for arc treatment

Given that we find an improvement in plan quality with the number of gantry directions, this may be extrapolated to arc treatments, which involve an even larger number of gantry angles. In fact, it is reasonable that the same number of segments, distributed over a larger angular range, should create better quality plans, since the number of degrees of freedom is increased. However, this may meet a limit for non-dedicated planning systems, since the inversion algorithms are sometimes unable to cope with a large number of gantry angles and few (or only one) segment per beam. For our example of only up to 18 beams, this does not seem to be the case. In particular, the 7XU beam plans were less sensitive to segment number of 18 beams, which may indicate also that FFF beams may be well suited for arc treatments.

### 6. Comparison with previous studies

A number of planning studies have compared flat and FFF plans for IMRT or VMAT. It is generally found that equal quality plans can be achieved using both modalities (e.g., [18]–[19]), a finding we confirm if a sufficiently high number of segments is available for the optimization. To our knowledge, no systematic study has been performed on the sensitivity of flat and FFF plans to a decrease in segment number. We find that the FFF beams are generally more sensitive to a decrease in the number of segments, which is physically plausible particularly for relatively large target volumes like in head-and-neck cancer.

Unexpectedly, the weaker plan quality of FFF beams for low numbers of segments is most strongly observed for plans with 7 or 11 gantry angles. If a large number of beams (18) is used, we generally obtain good or even better plan quality than for fewer beams in an otherwise identical scenario (energy and segment number), and less sensitivity to a reduction in segment number. For flat fields, Bratengeier et al. [20] have shown that plan quality and delivery time can be improved by increasing the beam number and reducing the number of segments. We observe a similar effect, which is even more pronounced in the case of FFF plans.

## Conclusions

We have presented a systematic analysis of plan quality for hypopharynx cancer planned with either 7, 11 or 18 beams and flat 6 MV vs. FFF 7 MV energy, for segments reduced from 70 to 25. As long as a sufficiently large number of segments is permitted, all planning scenarios give good results, independently on gantry angles and flat or FFF beams.

For smaller numbers of segments, plan quality decreases both for flat and FFF energies; this effect is stronger for fewer beams and for FFF beams. For low segment numbers, FFF plans are generally worse than the corresponding flat beam plans, but they are less sensitive to a decrease in segment number if many gantry angles are used (18 beams); in this case the quality of flat and FFF beams remains comparable even for few segments.
